# Extending Birthday Paradox Theory to Estimate the Number of Tags in RFID Systems

**DOI:** 10.1371/journal.pone.0095425

**Published:** 2014-04-21

**Authors:** Masoud Shakiba, Mandeep Jit Singh, Elankovan Sundararajan, Azam Zavvari, Mohammad Tariqul Islam

**Affiliations:** 1 Department of Electrical, Electronic and Systems Engineering, Faculty of Engineering and Built Environment, Universiti Kebangsaan Malaysia, UKM Bangi, Selangor, Malaysia; 2 Center for Software Technology and Management, Faculty of Information Science and Technology, Universiti Kebangsaan Malaysia, UKM Bangi, Selangor, Malaysia; University of Nottingham, United Kingdom

## Abstract

The main objective of Radio Frequency Identification systems is to provide fast identification for tagged objects. However, there is always a chance of collision, when tags transmit their data to the reader simultaneously. Collision is a time-consuming event that reduces the performance of RFID systems. Consequently, several anti-collision algorithms have been proposed in the literature. Dynamic Framed Slotted ALOHA (DFSA) is one of the most popular of these algorithms. DFSA dynamically modifies the frame size based on the number of tags. Since the real number of tags is unknown, it needs to be estimated. Therefore, an accurate tag estimation method has an important role in increasing the efficiency and overall performance of the tag identification process. In this paper, we propose a novel estimation technique for DFSA anti-collision algorithms that applies birthday paradox theory to estimate the number of tags accurately. The analytical discussion and simulation results prove that the proposed method increases the accuracy of tag estimation and, consequently, outperforms previous schemes.

## Introduction

Radio Frequency Identification (RFID) systems are a fast and reliable technology for identifying tagged objects by transmitting RF signals. As in the case of other identification technologies, such as barcodes, the main idea of an RFID system is to identify objects uniquely. An RFID system enjoys several technical advantages over barcodes, the most important of which is simultaneous identification, in contrast to sequential barcode reading.

As mentioned previously, the identification process in an RFID system is based on transmitting RF signals. Hence, in identifying multiple tags at the same time, there is a probability of collision among the signals that tags emit. When a collision occurs, the reader cannot identify the tags involved in the collision, and the identification process fails. As a result, the identification process has to repeat until all tags in the interrogation zone of the reader are identified successfully. Therefore, collision increases the identification time and energy consumption in the tag identification process.

Nowadays the application of RFID systems is increasing sharply, and RFID applications have dominated in almost all fields of science, such as social science [1], animal tracing [2], [3], health care [4], supply chain management [5], [6], and manufacturing [7]. In order to expand the application of RFID systems and increase their reliability, technical issues in RFID technology that delay identification and waste time and energy have to be considered.

Several anti-collision algorithms have been proposed to avoid the abovementioned situation and decrease the probability of collision. Anti-collision algorithms are generally categorized into ALOHA-based [8]–[16] and tree-based [17]–[23]. In ALOHA-based anti-collision algorithms, the reader allocates to the tags a frame involving the numbers of reading time slots, and each tag randomly selects a slot for transmitting its ID to the reader. The maximum performance of ALOHA-based anti-collision algorithms is obtained when the number of allotted slots is selected based on the number of tags. In contrast, in tree-based anti-collision algorithms the optimal number of branches depends on the number of unread tags [24]. Since primary knowledge of the number of tags is not available to the reader, we need to apply estimation methods to approximate the number of tags. Consequently, the accuracy of the applied tag estimation method has a direct effect on the performance of the RFID tag identification process.

In this paper, we propose a new tag estimation method based on birthday paradox probability theory. The results of applying the proposed method indicate higher accuracy and lower estimation error. Consequently, an accurate estimation results in a high performance tag identification process.

The rest of this paper is organized as follows. In the next section, we evaluate related work and discuss previous estimation methods. In section 3, the birthday paradox tag estimation method is presented in detail, followed by the results and discussion in section 4. Finally, we conclude in section 5.

### Evaluation of Previous Tag Estimation Methods

**Figure 1:**
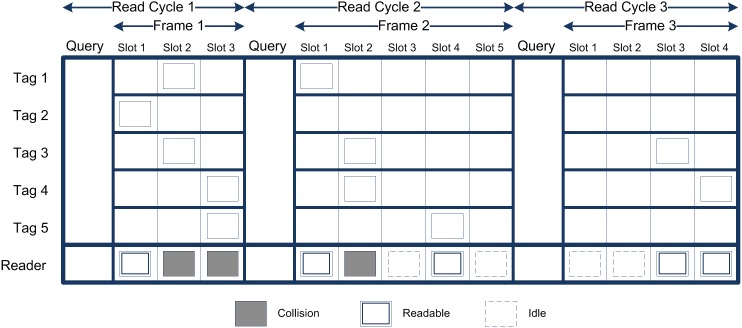
Dynamic Framed Slotted ALOHA Tag Identification Process.

In the ALOHA-based tag identification process, the reader first sends the tags a query with a frame size. Next, each tag in the interrogation zone of the reader selects a slot randomly in the range of the frame size. Then the reader scans the slots one by one. After a read cycle, in which all the slots are scanned, the reader provides a number triple, such as <*C_0_*, *C_1_*, *C_K_*>, which represents the number of idle slots, successful slots (slots with one tag), and collision slots (slots with more than one tag), respectively. Now, based on the number triple and knowledge of the frame size (*N*), the number of unread tags is estimated to determine the optimal frame size for the next read cycle. To obtain the general view of the identification process Figure 1, as an example, shows the DFSA anti-collision algorithm. As illustrated in the Figure 1, in the first read cycle, the reader sends the tags a query with a frame size (*N = 3*). Then all the tags select a slot randomly. Next, the reader scans all the slots one by one. After the first read cycle, we have a triple number as: *C_0_ = 0*, *C_1_ = 1*, and *C_K_ = 2*. Now based on the triple numbers and the first frame size we estimate the next frame size as *N = 5*. In the next read cycle only the tags involved in the collision response to the reader's next query. In this example, tag 2 successfully identified in the first read cycle, and not responses in other cycles. This process will continue until all tags be identified successfully. In the following, we explain some previous tag estimation methods and evaluate them critically.

### i. Lower Bound Tag Estimation Method

The lower bound tag estimation method is based on a simple assumption proposed by Vogt in 2002 [25]. Following Vogt’s suggestion, we assume that at least two tags are involved in a collision, so the number of tags is computed easily using Eq. (1).
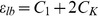
(1)


The lower bound is the simplest tag estimation method with very low computational requirements, just a simple addition and multiplication. Moreover, the lower bound method is very simple to implement, since it just multiplies a static number by the number of collisions. However, when the number of tags increases, the accuracy sharply decreases. This method is no longer used in ALOHA-based anti-collision algorithms owing to its low accuracy, but it is recommended for the lower tag range [26].

### ii. Schoute Tag Estimation Method

This method was proposed by Schoute et al. in 1983 [11]. In this method, the authors calculate the a posteriori expected value of a number of tags involved in a collision slot. They demonstrate that, on average, 2.39 tags are a constant value for collision slots. Thus, Schoute estimates the number of tags simply by using Eq. (2).
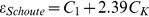
(2)


Just as with lower bound, the Schoute tag estimation method has low computational requirements and shows good performance in the low range of tag numbers. Schoute provides a more accurate estimation than the lower bound, but its accuracy falls sharply, when the numbers of tags are increased, because Schoute also estimates the number of tags statically.

### iii. Idle Slot Tag Estimation Method

Khandelwal et al. proposed this method, based on the probability of the idle slots occurring, in 2007 [27]. As shown in Eq. (3), when the number *C_0_ = 0* (there is no idle slot), the equation cannot be applied, so the authors used the lower bound estimation method in this case.
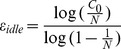
(3)In Eq. (3), *N* and *C_0_* represent the frame size and the number of idle slots respectively.

The idle slot method shows a higher accuracy in comparison with Schoute and lower bound; it dynamically estimates the number of tags when that number increases. However, the computational requirements necessitated by fractions and logarithms are much higher than those in Schoute and lower bound.

### iv. Chebyshev’s Inequality Tag Estimation Method

Applying Chebyshev’s inequality as a method for estimating the number of tags was proposed by Vogt in 2002 [25], [28]. Based on this theory, Vogt proposed an estimation function that uses the distance between the read result *C* and the expected value vector to determine the value of *n* for which the distance becomes minimal. Vogt denotes this estimation function by 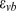, defined in Eq. (4).
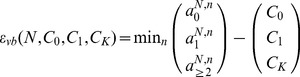
(4)where *N* represents the frame size, the variables (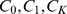) are the results of the read cycle (the number of slots captured by the reader), and the variables (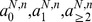) correspond to the expected value of the idle slots, successful slots, and collision slots, respectively, calculated as in Eq. (5).



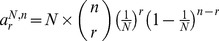
(5)The accuracy level of Chebyshev’s inequality is high and it changes dynamically with increasing numbers of tags, but it has the highest computational requirements, because it involves recursion.

### V. Bayesian Tag Estimation Method

Applying Bayes’ principles to a multi-access system seems not to be novel. Rivest suggested a Bayesian transmission approach for a slotted ALOHA broadcast system [29]. According to Rivest’s research, Floerkemeier also used the Bayesian transmission approach to an ALOHA RFID system [30]. These types of research basically consider ways to monitor system transmission. However, the tag estimation approach was not considered in such work.

Bayesian tag estimation was suggested in 2010 by Wu and Zeng, who see *n* as a random variable [31]. They also update the previous distribution of *n* through the posterior distribution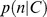 The tag quantity of Bayesian estimation is as in Eq. (6).
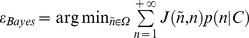
(6)where 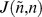 is a risk function and 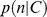 is the probability of *n*, given event *C*. The study tag range, the set 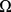, is as in (7).
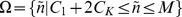
(7)where Wu and Zeng imagine that M is the optimal number of tags that an RFID system can read. In addition, they propose three different evaluations employing three types of risk functions. The Bayesian tag estimation method offers the highest accuracy among the proposed methods, but this method also suffers from high computational requirements resulting from recursion.

Table 1 summarizes and compares the characteristics of the abovementioned tag estimation methods.

**Table 1:** A short summary of the existing tag estimation methods’ characteristics.

Tag Estimation Method	Status	Accuracy	Computational Requirements
**Lower bound**	Static	Low	Low (addition, multiplication)
**Schoute**	Static	Low	Low (addition, multiplication)
**Idle Slots**	Dynamic	High	High (fractions and logarithms)
**Chebyshev’s Inequality**	Dynamic	High	Very High (recursion)
**Bayesian**	Dynamic	High	Very High (recursion)

### Methodology

As discussed in section 2, different researchers apply various mathematical rules and probability principles to achieve the optimal estimation of the number of RFID tags. In this section, we explain birthday paradox probability theory and propose a new tag estimation method by applying the extension of the birthday paradox to RFID systems.

#### Birthday paradox (problem) probability theory

In probability theory, the birthday paradox (problem) looks for the probability that at least two people have the same birthday in a given group of n randomly selected people. This theory involves the two assumptions that each day of the year is equally probable for a birthday, and that all the birthdays are independent [32].

We are not interested in the probability that at least two people share the same birthday. Therefore, we extend the birthday paradox theory to define a new problem. How many different birthdays on average will a group of *n* people have? To solve this problem, suppose that we have a random group of *n-1* people and that, on average, such a group has *E_n-1_* different birthdays. Now, if one person joins the group, the probability that this person’s birthday matches that of someone in the previous group is *E_n-1_/365*, and the probability that the person has a unique birthday is *1-E_n-1_/365*. Consequently, the value of *E_n_* can be calculated as in Eq. (8).
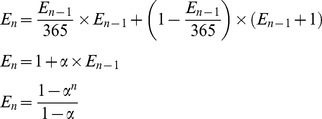
(8)where 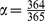.

The next interesting extension of the birthday paradox happens when *m* people in a group of *n* people share a common birthday; we call that birthday an *m*-birthday. We are interested in knowing the average number of *m*-birthdays in a group of *n* people. In other words, we are looking for the average number of days with exactly *m* births. We denote the average number of *m*-birthdays in a group of *n* people as *f_n_^m^* Based on the previously described method; *f_n_^m^* is calculated as in Eq. (9).
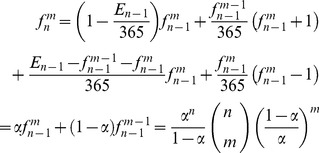
(9)


Based on Eq. (9), the average numbers of days with no birth and with only one birth for a randomly selected group of *n* people are as shown in Eq. (10) and Eq. (11), respectively:
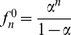
(10)

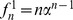
(11)


**Figure 2:**
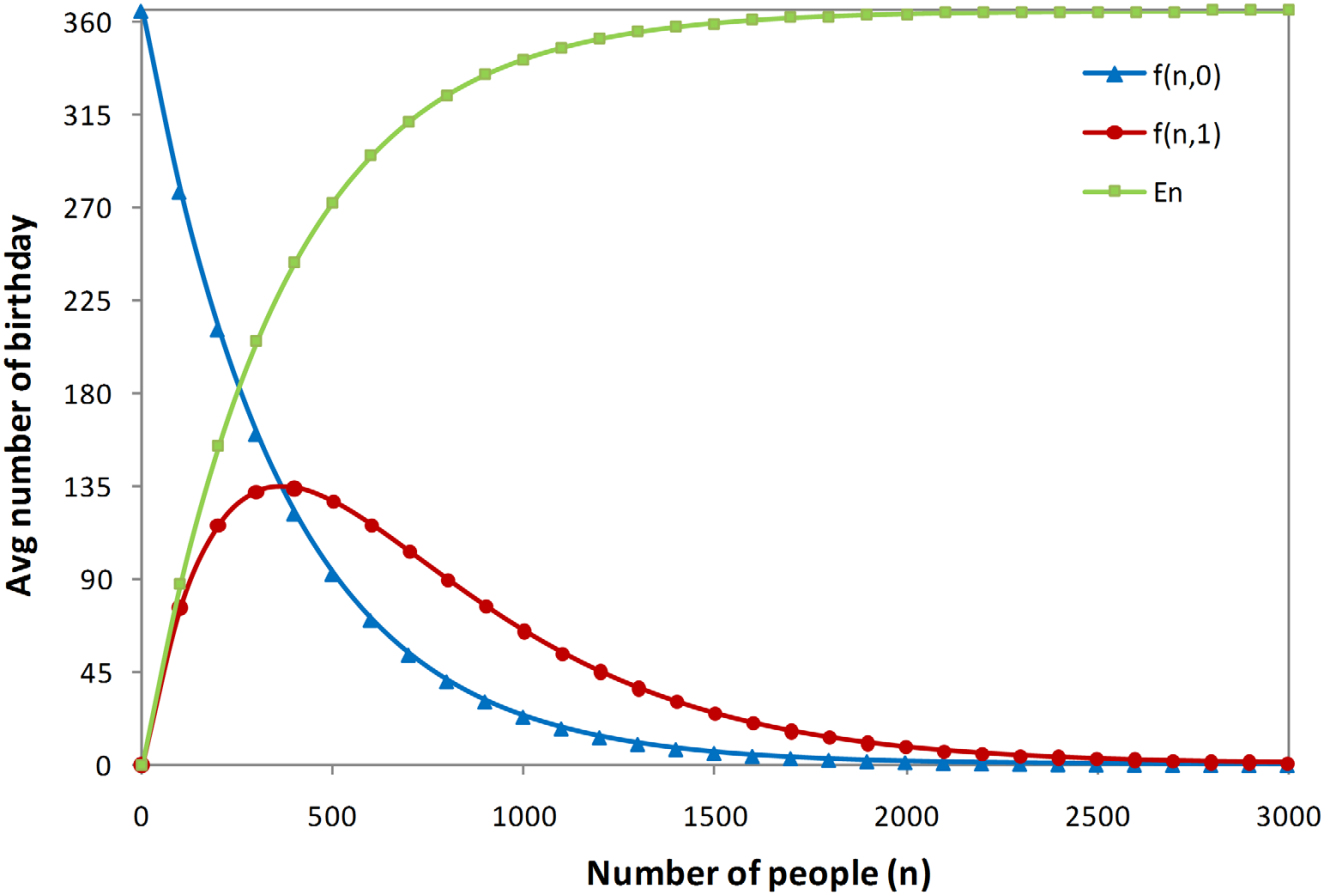
Average number of different birthdays as the number of people increases.

Therefore, based on Eq. (8) and Eq. (9), the average numbers of different birthdays a group of *n* people will have is given by Eq. (12). Figure 2 illustrates the behavior of *f_n_^0^*, *f_n_^1^*, and *E_n_* as the number of people increases.
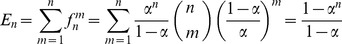
(12)


#### Birthday paradox-based tag estimation method

In the previous sub-section, we explained the birthday paradox and its extensions in detail. Now in this sub-section we want to apply the extension of the birthday paradox to RFID systems to estimate the number of tags. If we replace the people by tags and the days by slots in the birthday paradox, we can estimate the number of tags using Eq. (12), where 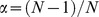.

We have already mentioned that there are three different situations for slots in RFID systems. First, there may be no tag in a slot, in which case the slot is an idle slot (no birth on that day, a *0*-birthday); second, when the slot is successful, only one tag transmits its ID to the reader (only one birth on that day, a *1*-birthday); and third, when more than one tag transmit their signals at the same slots (more than one person share same birthday, a *m*-birthday, *m>1*), there is a collision. We denote the three different situations as *C_0_*, *C_1_*, and *C_K_*, respectively. Therefore, based on the extension of birthday paradox, we have:
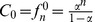
(13–a)

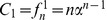
(13–b)

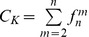
(13–c)

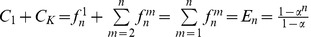
(13–d)


Hence, in RFID systems, the values of *C_0_*, *C_1_*, and *C_K_* are obtainable after any read cycle, and based on the known frame size, the value of 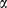 is available, so after any read cycle we can estimate the number of tags (*n*) by applying Eq. (13-a) as in Eq. (14).
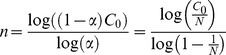
(14)


As illustrated in Eq. (14), the birthday paradox-based tag estimation method by using empty slots is proved by Eq. (3), which represents the idle slots tag estimation method [27]. Consequently, extension of the birthday paradox theory is a valid method for estimating the number of tags. As we have discussed, estimating the number of tags based on idle slots is not accurate enough when there is a large increase in the number of tags. When the number of tags is greatly increased, the value of *C_0_* goes to zero, and the estimation method fails. Hence, we propose a new estimation method based on *E_n_,* the number of collision slots plus the number of successful slots. According to Eq. (13-d), we have Eq. (15):
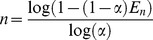
(15)


In previous studies, the communication channel between the tags and the reader is assumed to be error-free. Based on this assumption, the triple values of *C_0_*, *C_1_*, and *C_K_* are also supposed to be accurate. However, the real RFID communication environment is error prone, including what are known as capture effects. The capture effect is the correct and reliable identification of RFID tags when there is a probability of collision occurrence. In the real-world RFID system, the reader cannot provide accurate values for *C_1_* and *C_K_*. Since the reader cannot distinguish a collision with a transmission error, the value of *C_1_* and *C_K_* are underestimated and overestimated, respectively. Therefore, previous tag estimation methods fail to provide an accurate estimation in real-world RFID systems [33], [34]. Although the numbers *C_1_* and *C_K_* are sensitive to an error-prone channel, the sum of *C_1_* and *C_K_* (*E_n_*) is accurate and not sensitive to errors, because underestimating one of them (*C_1_* or *C_K_*) always results in overestimating the other and compensates for the error. As illustrated in Eq. (15), we estimate the number of tags base on *E_n_*, thereby guaranteeing accuracy in error-prone channels (real-world RFID system) and providing a high-performance identification process by allocating the optimum frame size and reducing the numbers of idle and collision slots.

As illustrated in the algorithm below, in the first step of tag estimation, all the existing tags are distributed into an initial frame (given slots) independently based on a uniform function [35], [36]. After all tags distribute and select a slot randomly, all slots are scanned one by one, and the numbers *C_1_* and *C_K_* are recorded by a reader. Hence, the tags are distributed using a stochastic function; there is a possibility of obtaining different values for *C_1_* and *C_K_* in different experiments. Thus, to obtain the optimal values for collision and successful slots, we repeat the simulation 500 times and compute *C_1Avg_* and *C_KAvg_*, the average number of successful and collision slots, respectively. Then, by calculating *E_n_* and *a* and applying Eq. (15), we obtain the estimated number of tags.

As we mentioned, the main objective of tag estimation methods is to estimate the number of tags in a more accurate manner to increase the performance of the identification system. Therefore, to show the effect of the proposed estimation method on the efficiency and performance of the tag identification process, different estimation methods are applied to a Dynamic Framed Slotted ALOHA (DFSA) anti-collision algorithm in the next section, and their performance is compared.

In a simple DFSA anti-collision algorithm, the reader starts the identification process by transmitting a query along with the initial frame size to all the tags in its interrogation zone. After any read cycle, based on the number of collision slots and the pre-defined thresholds, the reader dynamically changes the frame size. If the number of collision slots is greater than, less than, or between the thresholds, the reader increases, decreases or remains on the last frame size, respectively.

**Figure 3:**
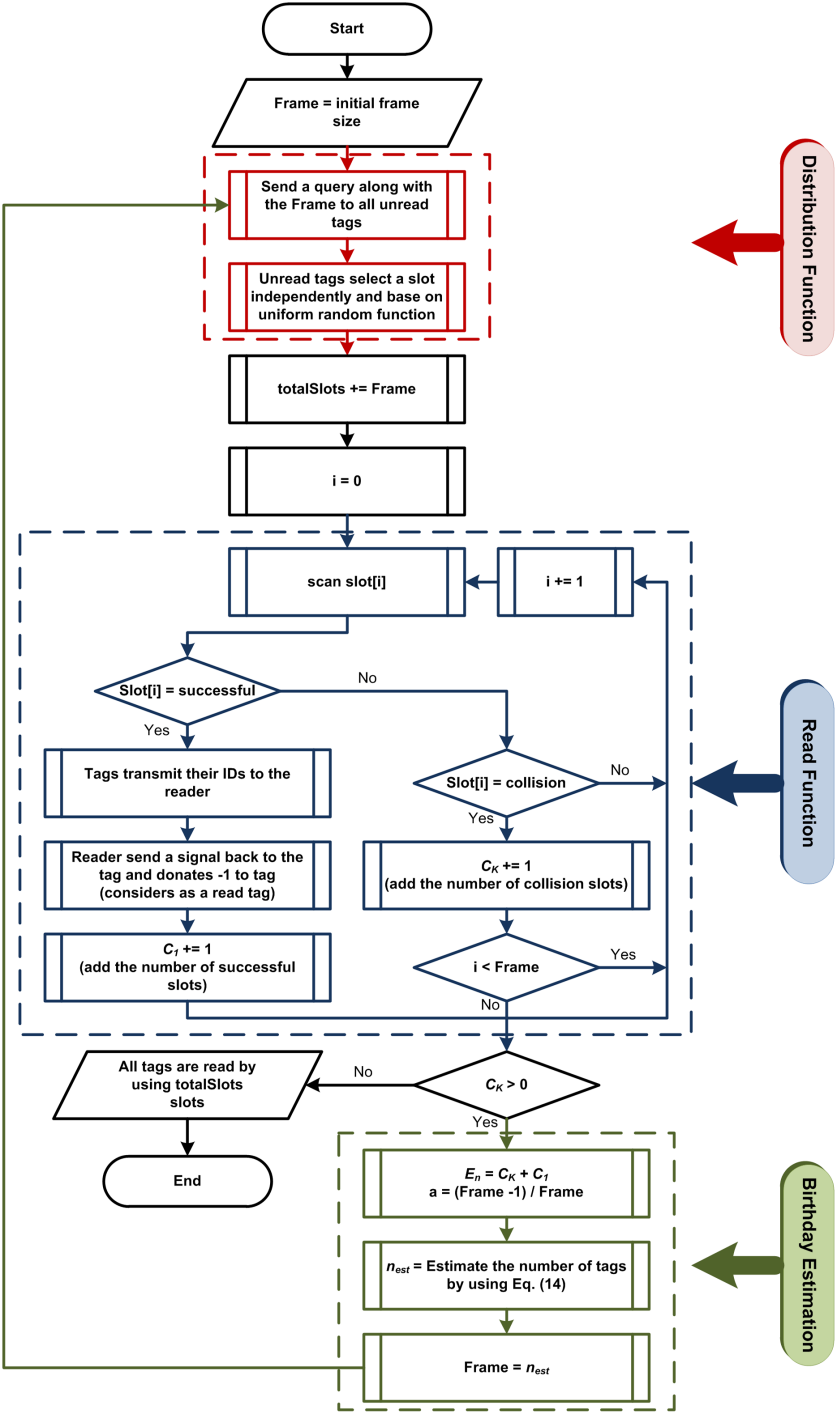
Applied DFSA anti-collision algorithms.

Another approach to the DFSA determines the next frame size based on the number of unread tags, so the reader determines the next frame size based on the tag estimation results. As demonstrated at the Figure 3 (flowchart), there are three major steps in the RFID tag identification process. At the first step, the reader sends a query along with the frame size to the all tags in its interrogation zone. Then all the tags select a slot randomly. This process, called distribution step.

At the second step, the reader scans the slots one by one, counts the number of successful and collision slots, and reads the successful tags.

Finally, at the third step, based on the frame size and the returned value of *E_n_* from the second step, the number of unread tags is estimated and allocated as the new frame size which is called as estimation function.

After approximation the number of tags, an optimal frame size is determined, and the algorithm starts from the first step by the new frame size. This process continues until there is no collision slot (all tags are successfully read).

The main objective of RFID systems is providing a fast identification process. In the application with the huge number of tags, the probability of collision occurrence will increase sharply. Hence, to minimize the collision occurrence, and maximize the performance of the RFID identification process, as previously mentioned, we need to obtain the optimal frame size based on the number of tags. Since the exact number of tags is not available, we need to approximate the number of existing tags. More accurate approximation, will result in minimum collision occurrence probability, which cause a fast identification process.

Based on the description above, all approximation methods follow special mathematical model to estimate the number of tags. Based on the extended birthday paradox model we estimate the number of tags accurately, and after precise approximation we can determine an optimal frame size and increase the performance and guarantee the fast identification process and achieve to the objective of the RFID systems.

Mathematical models have a direct role in estimating the number of tags, and the knowledge of the number of tags is essential to determine the optimal frame size, which causes the fast identification process. Therefore, mathematical models after precise approximation, guarantee the fast identification process.

Figure 3 shows the DFSA anti-collision algorithms applied in this paper.

## Results and Discussion

The birthday paradox-based tag estimation method is fully described in the previous section. The following presents a comprehensive comparison between the proposed method and previous techniques.

### 

#### i. Accuracy and error rate

**Figure 4:**
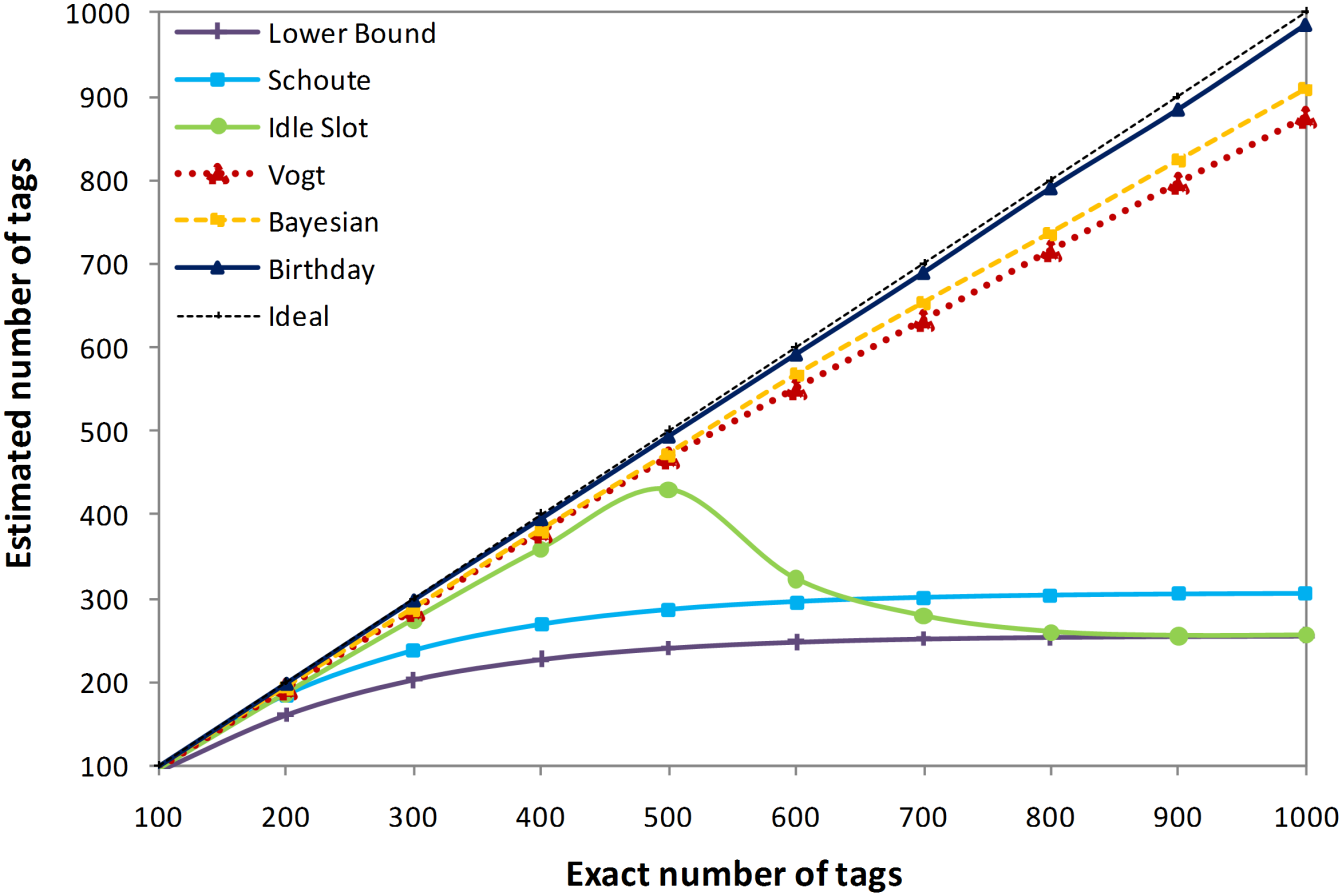
Comparison of estimated number of tags among different methods.

Figure 4 illustrates the estimation of the number of tags when the actual number of tags increased from 100 to 1000. The frame size is set to 128 for all methods. As shown in Figure 4, as the number of tags increases, the accuracy of the lower bound and Schoute estimation methods decreases and provides a poor estimation for the high number of tags, insofar as they estimate the number of tags as 255 and 305 for the actual 1000 tags, respectively. However, the idle slot presents a good accuracy, when the number of tags rises the number of C_0_ goes to Zero and the idle slot tag estimation applies the lower bound method so the accuracy fall down. Vogt, and Bayesian estimation methods show accurate results, however the birthday paradox-based tag estimation method provides a higher level of accuracy and estimates almost exactly the correct number of tags.

The main objective of all RFID tag estimation techniques is to provide a more accurate estimation of the number of tags with a lower error rate. Hence, to achieve a clear comparison among the estimation methods, the error rate (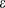) is defined as in Eq. (16), where 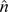 is the estimated number of tags and *n* is the actual number of tags [24], [31].
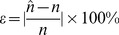
(16)


**Figure 5:**
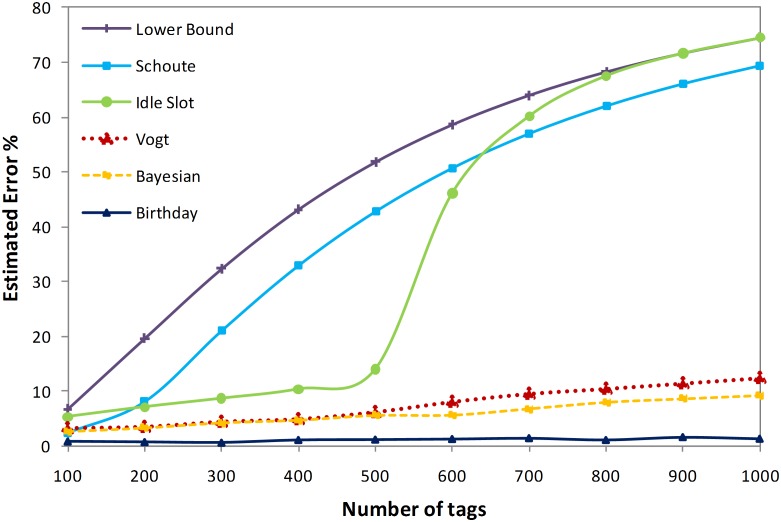
Comparison of estimation error among different methods.

Figure 5 demonstrates the error rate of different tag estimation methods when the number of tags is increased from 100 to 1000 and the initial frame size is set to 128. Based on Eq. (16), the lower bound estimation method shows a sharp increase in the rate of error, when the number of tags is increased. In the Schoute estimation method, for a small set of tags, an accurate estimation results with less than 5% errors recorded. However, the error rate increases clearly when the number of tags increases in comparison with the initial frame size, insofar as lower bound and Schoute show 74.45% and 69.47% tag estimation error rates, respectively. As already discussed the idle slot tag estimation method applies the lower bound when the number of *C_0_* equal to zero or one. Hence the accuracy of the idle slot falls down sharply when the number of tags increases, and presents a high level of error rate as illustrated in Figure 5. The Vogt and Bayesian estimation methods offer a smooth rate of error compared to the lower bound and Schoute estimations, with 12.46% and 9.22% average rates of error, respectively, in estimating the number of tags. As illustrated in Figure 5, the lowest rate of error belongs to the birthday paradox-based tag estimation method, which shows a 1.79% error rate on average, significantly increasing accuracy by decreasing the error rate.

In the section 2, we evaluated some existing tag estimation methods, and summarized their characteristics in Table 1. Now we want to evaluate the proposed method based on the same characteristics. Hence the estimated number of tags is not a static function of the captured slots, and it is dynamically changed based on the number of tags and the captured number of slots, therefore the proposed method is categorized at the dynamic methods. Furthermore, as illustrated in Figure 4, and Figure 5, the accuracy level of the birthday paradox-based tag estimation method, achieved to the highest level of accuracy with a lower rate of estimation error among the discussed methods; as a result the accuracy level of the proposed method is highest.

As demonstrated in Eq. (15) the computational requirement of the proposed method is the same as the idle slot tag estimation method. Both are based on the fractions and logarithms, so the method needs the high computational requirements. Table 2 summarizes the characteristics of the birthday paradox-based tag estimation method. As aforementioned, the proposed estimation method is a dynamic method with high computational requirements, which results highest accurate outputs.

**Table 2:** A short summary of the birthday paradox-based tag estimation method's characteristics.

Tag Estimation Method	Status	Accuracy	Computational Requirements
Birthday Paradox-based	Dynamic	Very High	High (fractions and logarithms)

Regardless of the high accuracy level, the strongest point of the proposed method is its compatibility to the error-prone channels which guarantees the highest level of accuracy in the real RFID communication channels. Estimating the number of tags by using the variable En covers the capture effects and performs highest results in real world RFID identification process. The highest accurate outputs and compatibility to error-prone environments cause the proposed method to be more advantageous in captured environments. The proposed method applicable and more advantageous at the application such as inventory management, warehouse management, secured document management, banknotes, postal packages and such as these applications where there are the huge number of tags to be identified

#### ii. Tag identification process

**Figure 6:**
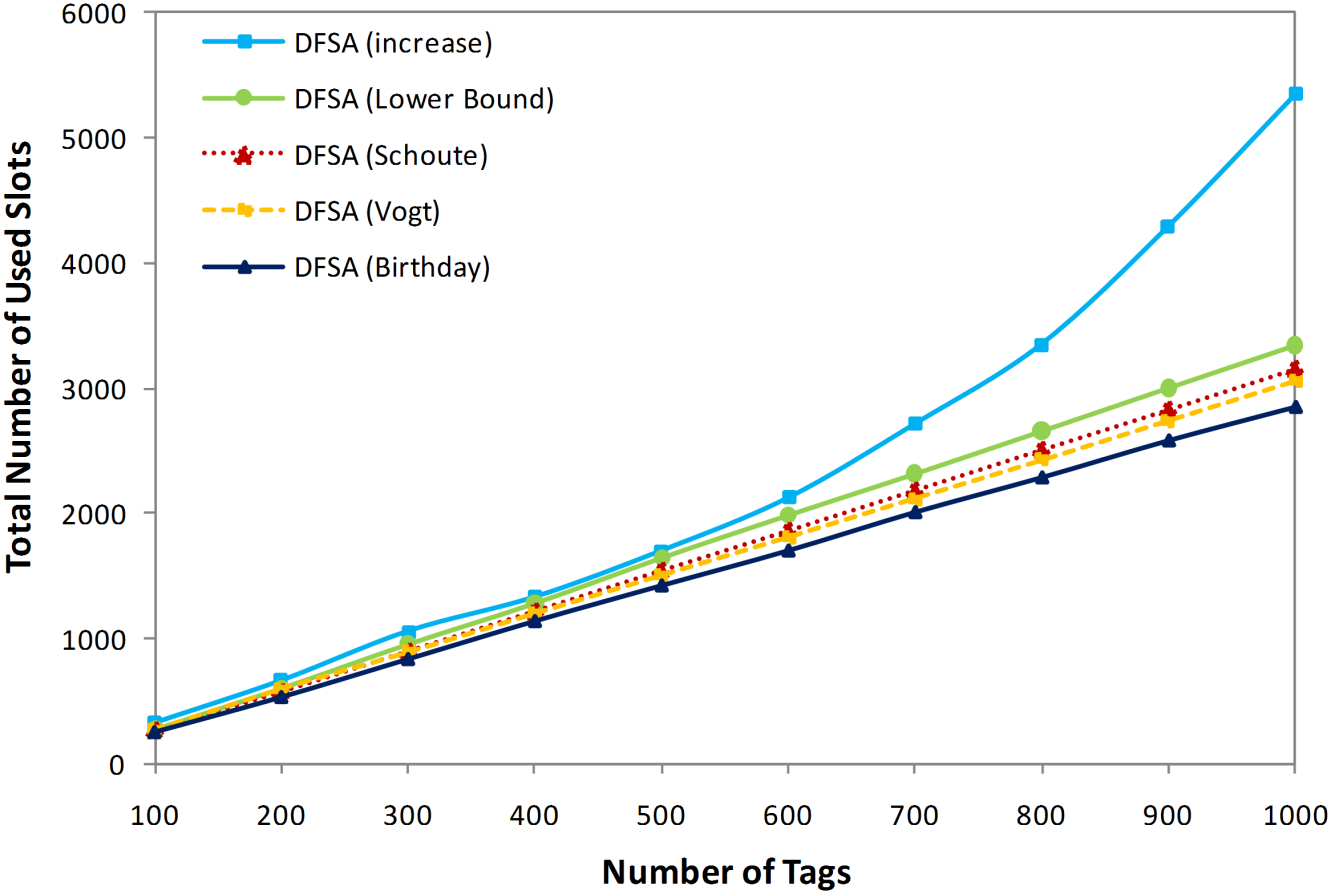
Total number of slots need to identify tags in different methods.

Just as in all identification technologies, the speed of object identification in RFID systems is a most important issue. Since, slots represent time duration in ALOHA anti-collision algorithms, identifying all tags successfully by using the minimum number of slots is the main goal. Therefore, to compare the effect of the higher accuracy of estimation methods on this important issue, Figure 6 shows the total number of slots used to identify a thousand tags for the different estimation methods applied to the same DFSA anti-collision algorithm.

As demonstrated in Figure 5, the birthday paradox-based tag estimation method presents the most accurate estimation outcome, so we expect it to identify tags more rapidly. In other words, based on the theoretical discussion, an accurate estimation method results in an identification process that uses the minimum number of slots to identify tags. As Figure 6 illustrates, the DFSA anti-collision algorithm applying the extension of the birthday paradox as an estimation method reduces the number of slots in use by 7.37%, 10.99%, 17.45%, and 87.74% in comparison with the increase, lower bound, Schoute, and Vogt, respectively, when there are 1,000 tags.

#### iii. Channel usage efficiency

As we have mentioned, the frame size in DFSA anti-collision algorithms should be selected based on the tag quantity. In this regard, there are numerous anti-collision algorithms that do not follow the standards, assuming that the durations of an idle slot, a collision slot, and a successful slot are identical. To obtain the maximum identification efficiency, the duration of slots can vary. In current RFID standards, such as ISO 18000–6 [34] and EPC global C1 Gen2 [37], an idle, a collision, and a successful slot duration have been set to be different. In these systems, a responding tag will first transmit its 16-bit random or pseudo-random number (RN16) to a reader in a slot, with three possible outcomes: terminating a slot ahead for no RN 16 information received at the reader, transmitting the tag's 64-bit electronic product code (EPC) after the RN 16 is correctly received, and not needing to transmit 64-bit EPC for an RN 16 colliding with others. Therefore, duration of the slots ranges from the most to the least as follows: successful, collision, and idle.

Here, we examine whether the maximum channel usage efficiency could be enhanced under the condition that an idle, a collision, and a successful slot duration are not identical. Suppose that *t_0_*, *t_1_*, and *t_K_* are an idle, a successful, and a collision slot duration, respectively. Then, channel usage efficiency can be found as in Eq. (17) [31], [38].
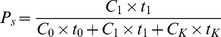
(17)


**Figure 7:**
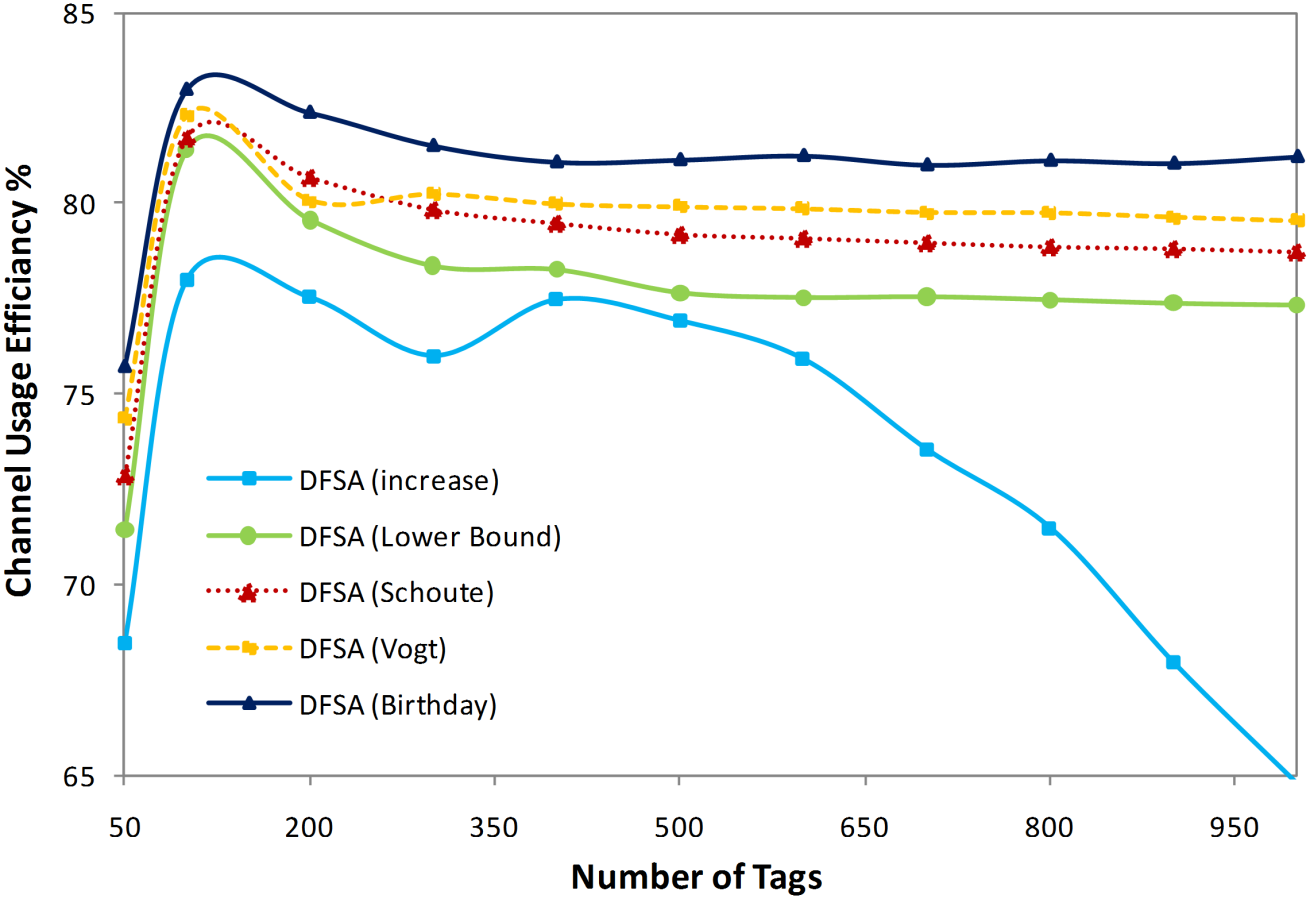
Channel usage efficiency of different methods.

Figure 7 shows the channel usage efficiency of various tag estimation methods applied to the same DFSA anti-collision algorithm when the number of tags has increased from 50 to 1,000. In the figure, the initial frame size is set to 128 and an idle, a successful, and a collision slot’s duration are set to 50, 400, and 50 µs, respectively. As illustrated in Figure 7, the highest channel usage efficiency belongs to the DFSA anti-collision algorithm using the birthday paradox-based tag estimation method and provides a monotonic range of around 81% channel usage efficiency.

#### iv.Average identification time

Providing an accurate estimation of the number of tags is the main objective of all the tag estimation methods. More accurate estimation results in an optimum frame size selection, which results in identifying the tags using the minimum number of time slots. Therefore, we can conclude that the main objective of all tag estimation methods is to provide a fast identification process in a shorter time.

As we have mentioned, we assume the initial frame size as 128, and suppose 50, 400, and 50 µs are the required times for idle, successful and collision slots, respectively. Hence, the identification time can be calculated using Eq. (18).
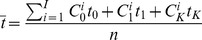
(18)


**Figure 8:**
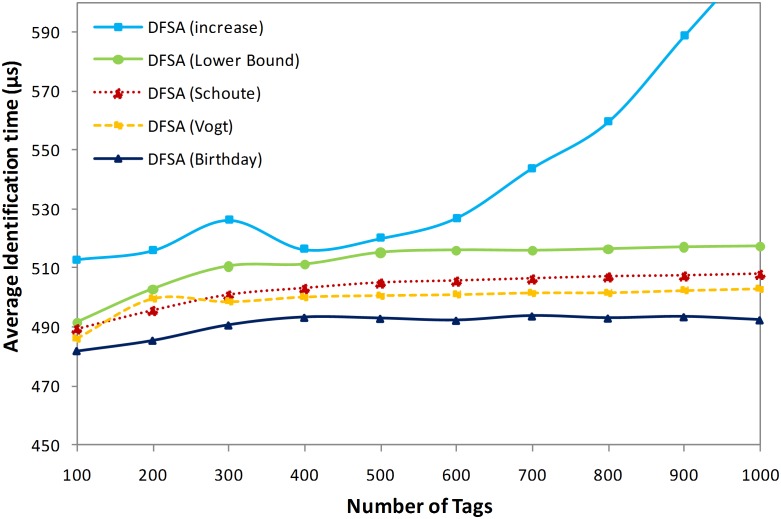
Average identification time for each tag in different methods.

Figure 8 shows the average identification time needed to identify a tag in the DFSA anti-collision algorithm applying different tag estimation methods. As is clear from the figure, the lowest average identification time to identify a tag belongs to the birthday paradox-based tag estimation method. Based on our aforementioned assumption regarding time slot duration, a successful slot requires 400 µs to be scanned completely by the reader to identify a tag. Therefore, based on Figure 8, the average wasted time to identify a tag in birthday paradox-based tag estimation is monotonically around 93 µs, which is a 9.7%, 12.9%, and 24.7% lower average wasted time in comparison with the Vogt, Schoute, and lower bound estimation methods, respectively.

## Conclusion

In this study, we proved that the proposed tag estimation method has better accuracy in comparison with previous estimation methods. The birthday paradox-based tag estimation method reduces the average tag identification time and increases the efficiency of the system by selecting the optimum frame size based on an accurately estimated number of tags. The high accuracy level, with an average error rate of 1.25%, in the proposed estimation method reduces the average wasted time in the tag identification process up to 24.7% in comparison with the lower bound method and increases the channel usage efficiency to 81%, a good performance.
